# Use of PET Imaging in Neuro-Oncological Surgery

**DOI:** 10.3390/cancers13092093

**Published:** 2021-04-26

**Authors:** Adrien Holzgreve, Nathalie L. Albert, Norbert Galldiks, Bogdana Suchorska

**Affiliations:** 1 Department of Nuclear Medicine, University Hospital, LMU Munich, 81377 Munich, Germany; Adrien.Holzgreve@med.uni-muenchen.de (A.H.); Nathalie.Albert@med.uni-muenchen.de (N.L.A.); 2 Department of Neurology, Faculty of Medicine and University Hospital Cologne, University of Cologne, 50937 Cologne, Germany; Norbert.Galldiks@uk-koeln.de; 3 Institute of Neuroscience and Medicine (INM-3), Research Center Juelich, 52425 Juelich, Germany; 4 Center of Integrated Oncology (CIO), Universities of Aachen, Bonn, Cologne, and Duesseldorf, 50937 Cologne, Germany; 5 Department of Neurosurgery, Sana Kliniken Duisburg, 47055 Duisburg, Germany

**Keywords:** PET imaging, glioma, glioblastoma, brain metastasis, meningioma, FET PET, somatostatin receptor, neurosurgery, neuro-oncological surgery

## Abstract

**Simple Summary:**

The use of positron emission tomography (PET) imaging in neuro-oncological surgery is an exciting field with thriving perspectives. Increasing evidence exists for amino acid-based PET to facilitate interpretation of imaging findings following therapeutic interventions in patients with glioma and brain metastases. In meningioma patients, radiolabeled somatostatin receptor ligands provide an improved tumor tissue visualization in lesions located in the vicinity of the skull base and differentiate between scar tissue and tumor recurrence. Moreover, they can be applied as an individual treatment option in recurrent atypical and anaplastic meningioma not eligible for further surgery and radiotherapy. With a focus on its clinical application, this review provides an overview of the emerging field of PET imaging in neuro-oncological surgery.

**Abstract:**

This review provides an overview of current applications and perspectives of PET imaging in neuro-oncological surgery. The past and future of PET imaging in the management of patients with glioma and brain metastases are elucidated with an emphasis on amino acid tracers, such as *O*-(2-[^18^F]fluoroethyl)-L-tyrosine (^18^F-FET). The thematic scope includes surgical resection planning, prognostication, non-invasive prediction of molecular tumor characteristics, depiction of intratumoral heterogeneity, response assessment, differentiation between tumor progression and treatment-related changes, and emerging new tracers. Furthermore, the role of PET using specific somatostatin receptor ligands for the management of patients with meningioma is discussed. Further advances in neuro-oncological imaging can be expected from promising new techniques, such as hybrid PET/MR scanners and the implementation of artificial intelligence methods, such as radiomics.

## 1. Introduction

In the evolving field of neuro-oncology, the management of both primary and secondary brain tumors can be improved by advanced imaging techniques. While conventional magnetic resonance imaging (MRI) reflects anatomical features, such as size and number of lesions, mass effect, perifocal edema, and contrast enhancement patterns, it cannot define the extent of metabolically active tumor tissue and intratumoral heterogeneity of the respective brain tumor. Both the increasing availability of elaborated tumor genetic analyses, such as next-generation sequencing as well as emerging new treatment options for primary and secondary brain tumors, including refined radiotherapy techniques and immunotherapeutic approaches, have led to a rising necessity of additional metabolic imaging techniques.

Positron emission tomography (PET) has proven to be a valuable tool in oncological settings since its propagation in the late 1970s. The basic principle of PET imaging relies on the so-called annihilation coincidence detection: while an intravenously injected isotope passes through the tissue, it emits positrons, annihilating with electrons; the reaction leading to a simultaneous emission of two photons heading in exactly opposite directions. A ring of detectors around the patient registers this reaction, respectively a projection of the line between the two photons. The resulting data of several projections from various coincidence lines are then reconstructed into three-dimensional images [1,2]. The majority of radiolabeled PET tracers are cyclotron-generated; the median half-life of F-18 is around 110 min.

The most common and widely used tracer in whole-body oncological imaging is the F-18-labeled glucose analog 2-[^18^F]fluoro-2-deoxy-D-glucose (^18^F-FDG). However, it has only limited clinical value in neuro-oncology due to the poor contrast between neoplastic and normal brain tissue. In contrast to ^18^F-FDG uptake, protein metabolism is clearly elevated in brain tumor cells compared to healthy brain tissue, making radiolabeled amino acids, such as [^11^C]-methyl-L-methionine (^11^C-MET), *O*-(2-[^18^F]fluoroethyl)-L-tyrosine (^18^F-FET), 3,4-dihydroxy-6-[^18^F]-fluoro-L-phenylalanine (^18^F-FDOPA), or ^18^F-fluciclovine (^18^F-FACBC) important targets for brain tumor imaging [3,4,5]. In correspondence to its increased availability, a high number of studies, including multicenter trials, have shown that amino acid PET (AA-PET) provides additional information on the metabolic properties of brain tumors. Furthermore, joint cooperatives, including the Response Assessment in Neuro-Oncology (RANO) Working Group, the European Association of Neuro-Oncology (EANO), the European Association of Nuclear Medicine (EANM), and the Society of Nuclear Medicine and Molecular Imaging (SNMMI), have issued guidelines recommending the implementation of AA-PET for purposes of differential diagnosis, treatment planning, and the differentiation of tumor relapse from treatment-related changes [5,6]. Additionally, the RANO group provided evidence-based recommendations for using PET imaging in patients with brain metastases, in meningioma patients, and for the planning and monitoring of radiotherapy in glioma patients [7,8,9].

**Figure 1:**
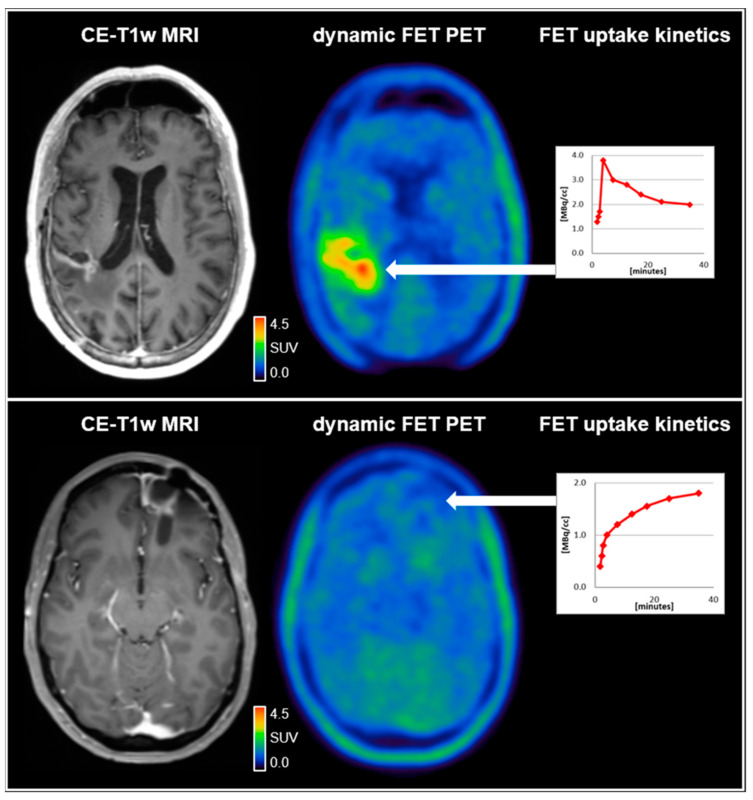
(**upper row**) A 59-year-old male patient diagnosed with an IDH-wild-type glioblastoma (WHO CNS grade 4). Following resection and chemoradiation with temozolomide, the contrast-enhanced MRI (CE-T1w MRI) suggested tumor relapse in the right parietal region 7 months after completing radiotherapy. Accordingly, the dynamic FET PET scan revealed pathologically increased FET uptake right parietal (TBR_max_, 4.2) and decreased time–activity curve; (**lower row**). A 37-year-old female patient diagnosed with an IDH-wild-type glioblastoma (WHO CNS grade 4). Following resection and chemoradiation with temozolomide, the contrast-enhanced MRI suggested tumor relapse in the left frontal region 7 months after completing radiotherapy. In contrast to the patient in the upper row, the FET uptake in the left frontal region was not pathologically increased (TBR_max_, 1.6) with a steadily increasing time–activity curve, indicating reactive treatment-related changes. SUV = standardized uptake value.

Amino acid uptake in both glioma and brain metastases is provided by large amino acid transporters (LAT) and has been shown to correlate with the density of LAT expression on the cell membrane surface in both entities [10]. In recent years, the relevance of AA-PET in the brain and spinal lesions has increased due to more refined imaging and postprocessing methods [2]. While the calculation of intralesional uptake relative to the healthy brain tissue, referred to as tumor-to-brain ratio (TBR), remains the standard in most centers, the analysis of tracer uptake dynamics is gaining further importance as it has been shown to enhance the diagnostic accuracy [11]. A reliable measurement of tracer uptake dynamics has predominantly been established for ^18^F-FET and, more recently, also for ^18^F-FDOPA [12]. For quantitative data analysis, mainly the dynamic parameters slope and time-to-peak (TTP) are calculated from time–activity curves (TAC). In glioma patients, dynamic parameters have been shown to correlate with the tumor grade according to older classification systems and, moreover, to molecular markers, such as mutations of the isocitrate dehydrogenase (IDH) gene [13,14]. Additionally, this technique allows differentiating between tumor progression and treatment-related changes in patients with glioma and brain metastases with high diagnostic accuracy [15,16]. An example illustrating the value of dynamic ^18^F-FET PET parameters for the differentiation between treatment-related changes and glioblastoma relapse is shown in Figure 1.

Apart from AA-PET tracers, several other radiopharmaceuticals have been developed for imaging in primary and secondary brain tumors, targeting hypoxia (^18^F-Fluoromisonidazole, ^18^F-FMISO), proliferation (3’-deoxy-3´-[^18^F]-fluorothymidine, ^18^F-FLT), and neuroinflammation (translocator protein, TSPO). Notably, in contrast to radiolabeled amino acids, all these tracers address specific metabolic conditions, and thus, their validity and usefulness for clinical purposes have yet to be shown in larger patient collectives.

In patients with meningioma, chelator-based tracers, such as [^68^Ga]DOTA-Tyr3-octreotide (^68^Ga-DOTATOC) and [^68^Ga]DOTA-D-Phe1-Tyr-3-octreotide (^68^Ga-DOTATATE), targeting somatostatin receptors are increasingly gaining importance in the management of recurrent or surgically poorly accessible tumors, both for the diagnostic and therapeutic use [7].

This review provides an overview of the current literature for using PET imaging in neuro-oncological neurosurgery. The first part focuses on using PET in patients with glioma and brain metastases, and the second part deals with applying PET in patients with meningioma.

## 2. PET Imaging in the Management of Patients with Glioma: Past and Future

Resection planning in glioma surgery involves two main goals: achieving maximal tumor removal while preserving functional tissue. Whereas advanced MRI techniques, such as diffusion tensor imaging and functional MRI (fMRI), allow for the evaluation of structural and functional connectivity in eloquent regions due to the infiltrative nature of glioma growth, the delineation of non-enhancing glioma tissue in areas with signal hyperintensity in T2-weighted MR images may be difficult based on conventional MRI alone. Visualization of areas with tumor infiltration using 5-aminolevulinic acid (5-ALA) is of value for intraoperative decision-making and has been repeatedly shown to increase the extent of resection rates [17]. While morphological imaging using different MRI techniques to determine the expected extent of fluorescence in preoperative resection planning has not proven to be of particular value, ^18^F-FET uptake was shown to be more sensitive to detect tumor tissue than intraoperative 5-ALA [18]. A recent study evaluating the outcome in glioblastoma patients undergoing 5-ALA-based tumor resection exceeding contrast enhancement borders on MRI has shown that the remaining ^18^F-FET PET volume was associated with the overall survival [19]. However, prospective data on the benefit of a combined resection planning relying on information provided by both 5-ALA and metabolic imaging is currently lacking.

In eloquently localized tumors without clear contrast enhancement and not eligible for complete resection, identifying the most malignant tumor parts representing intratumoral heterogeneity is essential for the planning of the surgical procedure [20]. Here, PET-guided biopsy has been recommended to improve the diagnostic yield and to avoid the biopsy of inconclusive tissue samples. Indeed, dynamic ^18^F-FET PET has been shown to detect aggressive tumor sub-volumes, and dynamic ^18^F-FET uptake parameters calculated from time–activity curves have shown to be an independent biomarker for clinical outcome [20,21,22].

Furthermore, the refinement of molecular sequencing techniques has, not only since the revision of the World Health Organization (WHO) Classification of Tumors of the Central Nervous System (CNS) in 2016, prompted increasing research interest in metabolic and genetic properties in brain tumors [14]. In particular, discovering mutations of the isocitrate dehydrogenase (IDH) gene as a pivotal point in gliomagenesis and its impact in terms of prognosis of glioma patients is of utmost importance in neuro-oncology [23]. It not only seems to indicate a diverse origin of glial tumors but also has been shown to be a prognostic marker [24]. In contrast to metabolic heterogeneity, molecular biomarkers, such as *IDH* mutation and O-6-methylguanine-DNA methyltransferase (*MGMT*) promoter methylation, have shown a homogeneous distribution within glial tumors [25]. Thus, the focus of metabolic imaging in glioma continues to shift from the identification of metabolic hotspots towards the non-invasive prediction of molecular tumor characteristics. Radiological features, such as tumor location and contrast enhancement patterns, have already been shown to provide a tool for the assessment of surgical benefit in glioma [26]. WHO grade II *IDH* mutant gliomas seem not only to evolve in less eloquent brain regions, but the affected patients also may have a greater benefit from maximal tumor resection [26]. This approach should provide an additional tool to stratify patients in high- and low-risk groups taking into account not only their age and performance score but also the anticipated benefit from surgical procedure.

It has been reported that parameters obtained from dynamic AA-PET acquisition are of clinical value for predicting molecular markers and the estimation of prognosis in patients with newly diagnosed glioma. A recent study identified a positive predictive value of 87% for the prediction of an *IDH* wild-type status in patients with newly diagnosed WHO II-IV glioma using dynamic ^18^F-FET PET data [27]. Similarly, a hybrid ^18^F-FET PET/MRI radiomics study in glioma patients identified an *IDH* mutation with an accuracy of 93% when combining static, dynamic, and textural ^18^F-FET PET data [28]. Parameters derived from dynamic ^18^F-FET PET analysis have additionally shown to be an independent predictor for overall survival in patients with astrocytic *IDH* mutant glioma, but not in oligodendrogliomas or *IDH* wild-type gliomas [29]. Subsequently, an association between dynamic PET parameters and overall survival also has been observed for patients with *IDH* wild-type glioma [21]. Nevertheless, in the light of the upcoming WHO CNS classification expected in 2021, the prognostic impact of parameters derived from dynamic ^18^F-FET PET for distinct tumor subgroups must be re-evaluated in further studies.

The calculation of biological tumor volume (BTV) in PET images, especially the uptake volume of radiolabeled amino acids, also seems to be of clinical value. Several studies have suggested that in most patients with newly diagnosed glioma, the BTV is considerably larger than the contrast-enhancing volume on MRI, which is valuable for neurosurgical resection planning [30].

Furthermore, another study highlighted the prognostic value of the BTV after resection in newly diagnosed glioblastoma patients [31]. Several studies, including prospective trials, have suggested that a BTV of more than 10 mL following microsurgery before initiation of chemoradiation is associated with shortened overall survival [31,32]. Moreover, parameters obtained from dynamic AA-PET were associated with outcome during and following radiotherapy, both in patients with newly diagnosed and recurrent glioma undergoing re-irradiation [31,33,34]. Accordingly, the survival benefit of an ^18^F-FET PET-based target volume definition compared to conventional MRI before re-irradiation of patients with glioblastoma relapse is currently evaluated within the NOA-10/GLIAA trial [35].

Additionally, BTVs calculated from AA-PET seem to be of value for the assessment of response to temozolomide chemoradiation and maintenance temozolomide chemotherapy in glioblastoma patients and in patients with predominantly non-enhancing WHO grade II glioma undergoing alkylating chemotherapy [36,37,38]. For example, MRI-based evaluation of treatment response in patients with eloquently located non-enhancing glioma treated with alkylating chemotherapy is hampered by the prolonged period until the response on MRI becomes apparent in terms of volume shrinkage on T2-weighted MR images [39]. In these patients, using ^18^F-FET PET for response assessment has provided a more accurate evaluation of metabolic response or failure to alkylating chemotherapy, allowing for a timely adjustment of treatment. Furthermore, both decrease in metabolically active tumor volume and absolute tracer uptake, as well as a combination of both values, were predictive for a prolonged progression-free survival [38].

## 3. Clinical Value of PET in Patients with Brain Metastases

The incidence of brain metastases occurring in approximately up to 20% of cancer patients is supposed to increase due to improved systemic treatment options and monitoring in patients with extracranial tumors. Apart from local treatment options, including microsurgery, external beam radiotherapy, and radiosurgery, there is increasing evidence for the efficacy of systemic treatment in brain metastases [40]. Due to the high sensitivity of conventional MRI, even for small brain metastases within the millimeter range, it is the method of choice to manage patients with brain metastases [41]. Notably, AA-PET detected 90% of all newly diagnosed brain metastases compared to ^18^F-FDG PET (21%). Nevertheless, it should be considered that its sensitivity in lesions smaller than 1 cm in diameter is diminished due to the lower spatial resolution of PET scanners [42,43]. Furthermore, although the uptake behavior was heterogeneous in metastases of different histological origins, AA-PET could not identify a characteristic pattern, which may help identify a particular primary tumor subtype [43]. Thus, more (neuropathologically confirmed) data on metabolic tumor properties of histologically different brain metastases are needed to provide a PET-based tool for predicting tumor origin.

With increasing incidence and availability of treatment strategies, including the heightened use of radiosurgical techniques, the differentiation between treatment-related changes, such as radionecrosis and brain metastases relapse, remains still challenging. However, this differentiation based on structural imaging alone is limited due to its low specificity for neoplastic tissue [44]. Several recent studies have evaluated the discriminative power of AA-PET in suspected radiation-induced changes compared to brain metastases relapse. The general observation was a significantly increased uptake in relapsing brain metastases compared to radiation-induced changes; the accuracy in providing a correct classification ranges from 77% to 90% [45,46,47]. In particular, the addition of dynamic evaluation of the ^18^F-FET uptake improved the diagnostic performance [15,45,48].

It has been demonstrated that radiomics using textural features obtained from metabolic imaging (e.g., gray levels, tissue entropy, spatial arrangement of single voxels) provides additional diagnostic information. Textural features extracted from MRI alone had a diagnostic accuracy for predicting radiation-induced changes versus tumor recurrence in previously irradiated brain metastases of 81%, while the accuracy of ^18^F-FET PET radiomics features was slightly higher (83%). Notably, the best discriminative power with an accuracy of 89% was achieved when the information of both modalities was combined [49].

Systemic treatment options based on immunotherapeutic approaches, such as checkpoint inhibitors used in patients with lung cancer or melanoma brain metastases, may impose the problem of treatment-related changes related to inflammation [41,50]. As a consequence, a misinterpretation of conventional MRI may cause premature discontinuation of effective therapy with a potentially negative impact on survival.

An initial pilot study in patients with melanoma brain metastases treated with ipilimumab suggested that amino acid PET using ^18^F-FET can identify checkpoint inhibitor-related pseudoprogression [51]. A subsequent case report revealed a reduction in metabolic activity on AA-PET in a patient undergoing targeted therapy for non-small-cell lung cancer brain metastases indicating tumor response, while MR findings remained unchanged [52]. A recent study in a larger series of patients (*n* = 40) with more than 100 brain metastases secondary to melanoma or non-small cell lung carcinoma treated with checkpoint inhibitors or targeted therapy combined with radiosurgery reported that ^18^F-FET PET provides important diagnostic information in terms of response assessment [53]. Moreover, in that study, metabolic responders on ^18^F-FET PET had a significantly longer progression-free survival in contrast to response determination based on contrast-enhanced MRI.

Another promising approach for the inclusion of metabolic imaging data for the response evaluation of checkpoint inhibitors and targeted therapies is using PET tracers designed to assess proliferation, such as ^18^F-FLT. The principle of ^18^F-FLT is based on phosphorylation of the fluorinated thymidine analog by the enzyme thymidine kinase 1 (TK1) and its consecutive entrapment within the tumor cell [52,54]. TK1 is predominantly expressed in highly proliferating cells. Thus, a heightened ^18^F-FLT uptake within the cell reflects an increased TK1 activity. A pilot study assessing the role of ^18^F-FLT PET imaging for treatment response assessment in patients with melanoma brain metastases treated with either targeted therapy or immunotherapy has shown a considerable reduction of ^18^F-FLT uptake in treatment responders compared to only minimal or no changes on the corresponding MR images [55].

## 4. Specific Somatostatin Receptor Ligand PET in Meningioma

Meningiomas constitute most primary brain tumors, about 80% being classified as WHO grade I, whereas WHO grade II and III meningioma are less common [56]. If indicated, microsurgical resection is generally the therapy of choice. Radiotherapy, including radiosurgery, which is predominantly used in the recurrent situation, may be preferred in small WHO grade I meningioma or in locations not eligible for complete neurosurgical resection [57]. Standard MRI is the imaging method of choice and usually shows a homogeneous contrast enhancement and a characteristic attachment to the dura mater, the dural tail sign [57].

On the molecular level, meningiomas may express various hormonal receptors for progesterone and estrogen, as well as specific somatostatin receptors (SSTR) [58,59]. SSTR expression is also often encountered in neuroendocrine tumors, and several tracers have been implemented based on radiolabeled SSTR ligands, usually compounds containing SSTR agonists, such as tyrosine^3^-octreotate (TATE) or the octapeptide octreotide (TOC) and a chelator, e.g., tetraxetane (DOTA), coupled to the short-lived radionuclide gallium (Ga-68) [60]. Recently, an F-18-labeled SSTR-targeted tracer with strong tumor uptake as well has been implemented, which could significantly facilitate a broader application of SSTR-targeted PET in neuro-oncology as an expensive on-site Ga-generator would no longer be required [61].

Since their development in the 1990s, SSTR agonists have been increasingly used in meningioma imaging [62]. The main indication for SSTR PET is identifying meningioma tissue, including the delineation of meningioma extent, especially in complex anatomical regions, such as the skull base or the orbital region, and with a special focus on the diagnosis of intraosseous infiltration [63,64]. For example, ^68^Ga-DOTATATE PET was able to identify meningioma out of 13 intraorbital symptomatic lesions with both specificity and sensitivity of 100% [65].

**Figure 2:**
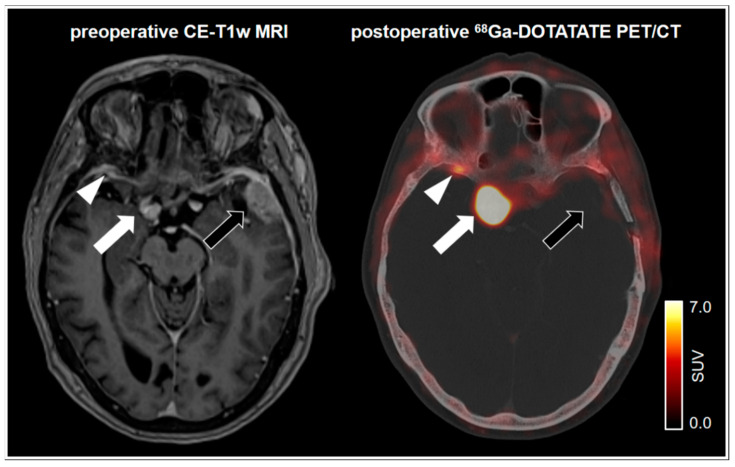
75-year-old female patient with a left frontotemporal transitional meningioma of the WHO grade 1. ^68^Ga-DOTATATE PET/CT shows no postoperative remnants of the left frontotemporal tumor (black arrow). Pronounced tracer uptake in the right parasellar region indicates a meningioma in correlation to the MRI (white arrow). Notably, a small focal uptake posterior to the right orbital region indicates an additional meningioma. In spatial correspondence, the MRI shows equivocal findings (white arrowhead). SUV = standardized uptake value.

Exact tumor delineation in complex anatomical regions, such as the skull base, is not only crucial for surgical considerations but also of crucial importance for radiotherapy planning. Inclusion of PET imaging for stereotactic radiotherapy planning was not only able to identify infiltrated tissue and provide information beyond bone windowing on CT and contrast enhancement on MRI, but also has led to a preservation of critical areas, such as the pituitary gland and the optic chiasm [66,67]. An example of MRI compared to postoperative ^68^Ga-DOTATATE PET revealing additional tumor tissue is shown in Figure 2. It should be noted that tumor delineation may be hampered in tumors located in the vicinity of the pituitary gland, which has an endogenously high SSTR expression. On the other hand, this phenomenon may positively control correct tracer application [68].

Following treatment, ^68^Ga-DOTATATE PET was able to differentiate between scar tissue and vital remnants or tumor recurrence with a specificity of 74% and a sensitivity of 90%, outperforming contrast-enhanced MRI [69]. Interestingly, while intraosseous meningiomas tend to have even higher uptake than extraosseous tumors, the accuracy of ^68^Ga-DOTATATE PET for differentiation between tumor progression and treatment-related changes was even higher in these lesions with a specificity of 100% and a sensitivity of 97% [70]. A recent case report suggested that SSTR PET helps to differentiate a possible metastatic lesion from meningioma tissue in a patient with a history of previous cancer [71].

Predominantly in patients with recurrent atypical and anaplastic meningioma not eligible for further surgical or radiotherapeutic interventions, the exchange of the radiolabeled isotope gallium-68 to lutetium-177 or yttrium-90 allows for peptide receptor-based radionuclide therapy (PRRT) [72]. Similar to SSTR-based imaging, PRRT has been first established in neuroendocrine tumors. Thus, most information on efficacy and toxicity is based on experience with neuroendocrine tumors [73]. The adverse event profile includes hematotoxicity, including leukopenia and myelodysplastic syndromes, nephrotoxicity, and gastrointestinal irritation, but is generally mild compared to adverse events related to other systemic treatment options. Several studies have been published on outcomes in recurrent tumors treated by PRRT, local tumor control rates ranging from 60 to 86%. However, the results of the different studies cannot be generalized due to different follow-up intervals, imaging procedures, choice of tracer, and application dose [74,75]. Nevertheless, the reported results are encouraging and allow for individualized treatment in patients with recurrent meningioma of the WHO grade II or III.

## 5. Conclusions

Magnetic resonance imaging, being widely accessibleand easy to interpret due to standardized protocols and assessment criteria, still remains the method of choice in neuro-oncology imaging. However, increasing evidence exists for amino acid-based PET to facilitate interpretation of imaging findings following therapeutic interventions in patients with glioma and brain metastases. In meningioma patients, radiolabeled somatostatin receptor ligands provide an improved tumor tissue visualization in lesions located in the vicinity of the skull base as well as for differentiation between scar tissue and tumor recurrence. Moreover, they can be applied as an individual treatment option in recurrent atypical and anaplastic meningioma not eligible for further surgery and radiotherapy. Further advances in neuro-oncological imaging can be expected from promising newer techniques, such as a combination of PET/MR imaging using hybrid scanners and the implementation of methods from the field of artificial intelligence.

## Data Availability

No new data were created or analyzed in this study. Data sharing is not applicable to this article.
